# Integrated genomic analysis identifies subclasses and prognosis signatures of kidney cancer

**DOI:** 10.18632/oncotarget.3294

**Published:** 2015-03-24

**Authors:** Yann Christinat, Wilhelm Krek

**Affiliations:** ^1^ Institute of Molecular Health Sciences, ETH Zurich, 8093 Zurich, Switzerland

**Keywords:** miRNA, cancer, clear-cell renal cell carcinoma, prognosis, TCGA

## Abstract

**Purpose:**

To define robust miRNA-based molecular classifiers for human clear cell renal cell carcinoma (ccRCC) subgrouping and prognostication.

**Experimental design:**

Multidimensional data of over 500 clear cell renal cell carcinoma (ccRCC) patients were retrieved from The Cancer Genome Atlas (TCGA) archive. Data analysis was based on a novel computational approach that selectively considers patients with extreme expression values of miRNAs to detect survival-associated molecular signatures.

**Results:**

Our *in silico* analysis unveiled a novel ccRCC-specific 5-miRNA (miR-10b, miR-21, miR-143, miR-183, and miR-192) signature able, when combined with information from conventional TNM staging and the age of the patient, to prognosticate ccRCC outcome more accurately than known ccRCC miRNA signatures or TNM staging alone. Furthermore, our approach revealed the existence of 6 distinct subgroups of ccRCC characterized by discrete differences in overall survival, tumor stage, and mutational spectra in key ccRCC tumor suppressor genes. It also demonstrated that *BAP1* mutations correlate with tumor progression rather than overall survival.

**Conclusion:**

Integrated analysis of multidimensional data from the TCGA archive allowed to draw a portrait of distinct molecular subclasses of human ccRCC and to define signatures for prognosticating disease outcome. Together, these results offer new prospects for more accurate stratification and prognostication of ccRCC.

## INTRODUCTION

Renal cell carcinoma (RCC) is a frequent malignancy affecting nearly 300,000 individuals worldwide [1]. The most common subtype is clear-cell renal cell carcinoma (ccRCC) which accounts for 70–80% of all renal malignancies [2]. Recent advances in the genomic analysis of ccRCC revealed extensive tumor heterogeneity [3, 4]. This poses a formidable challenge to complement the currently most accepted clinical decision making basis, the TNM staging [5] with molecular signatures derived from genomic analyses for prognostication and prediction of ccRCC outcome and response to therapy, respectively.

MicroRNAs (miRNAs) are a class of small non-coding RNAs that are increasingly considered as molecular biomarkers for tumor diagnosis, prognosis, and prediction [6]. In ccRCC, several miRNAs have been identified and associated with specific aspects of ccRCC biology. For example, a 11-miRNA signature has been suggested to distinguish between ccRCC and normal tissue [7] and other miRNA signatures have been linked to metastases, recurrence, prognosis, or RCC subtypes [8–14]. Remarkably, the overlap between these signatures is almost inexistent. A common methodology to these studies is to group patients into two categories based on pathological features—such as normal vs. tumor tissue, overall survival, tumor stage, or presence of metastasis—and to identify miRNAs that are differentially expressed. While this methodology is valid for binary variables such as presence of metastasis, it becomes inappropriate for continuous data such as overall survival or disease-free survival [15]. Also most studies rely on a rather small number of samples. With respect to the latter, the Cancer Genome Atlas (TCGA) Research Network recently provided a comprehensive molecular description of more than 500 ccRCCs and over 60 matching normal controls with respect to genomic alterations, RNA and proteomics signatures, DNA methylation profiles and clinical and pathological features [16]. Data were collected through several studies across more than ten different institutions, creating thus a robust, diverse, and unique dataset of ccRCC samples. Through *in silico* analysis of their comprehensive genomic data sets, they were able to identify four major patient clusters with different outcomes and characterized by a 4-miRNA signature [16].

Here we re-analyzed these TCGA datasets on ccRCC with a new computational approach and assessed its performance through the concordance index, avoiding thus the continuity issue associated with survival variables. Our analyses identified novel miRNA signatures linked to patient subclassification and survival. The latter, when combined with patient's age and conventional TNM-based staging information, allows prognostication of ccRCC with superior power over published molecular signatures or the TNM stage information alone.

## RESULTS

### Identification of miRNA clusters associated with overall survival in ccRCC

**Figure 1:**
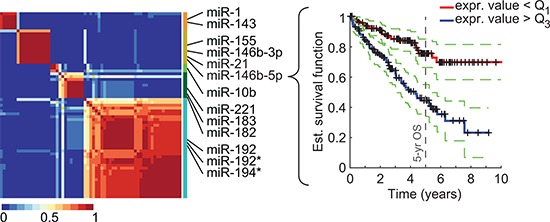
Identification of miRNAs associated to overall survival (OS) in ccRCC NMF clustering consensus map of the 65 identified miRNAs (training cohort) and Kaplan-Meier plot (validation cohort) for the most significantly OS-associated cluster representative, miR-146b-5p. Identified clusters (color-coded) and representative miRNAs are displayed on the right side of the map. Dotted lines represent the 5% confidence interval.

To identify miRNAs linked to patient survival, we used TCGA datasets of over 500 ccRCC patients that we split into training and validation cohorts based on the respective miRNA-sequencing technology, yielding 252 patients in the training cohort and 261 in the validation cohort. We then asked whether low or high levels of a given miRNA had a significant correlation with a patient's overall survival. For each miRNA, patients were first separated by expression level quartiles of the given miRNA. Then, the overall survival of the patient group characterized by low expression of the miRNA—below the first quartile—was compared to the survival of the patient group with high expression levels—above the third quartile—through a log-rank statistical test. Using this new methodology, we identified 65 miRNAs that were statistically linked to overall survival (pFDR < 0.1; Supplementary Table S1). Among them, 32 were also significantly associated to overall survival in the validation cohort (*p*-value of log-rank test < 5%). A clustering procedure revealed the presence of 5 distinct miRNA clusters, which were best represented by miR-21, miR-146b-3p/5p, and miR-155 for cluster 1, miR-1 and miR-143 for cluster 2, miR-10b for cluster 3, mir-194-3p and miR-192-3p/5p for cluster 4, and miR-182, miR-183, and miR-221 for cluster 5 (Fig. 1). Note that although published miRNA signatures overlap with our set of 65 miRNAs, none has miRNAs in more than 3 of the 5 identified clusters.

### miR-21, miR-10b, miR-143, miR-183, and miR-192 define a prognosis signature in ccRCC

A list of miRNAs that significantly affect survival is a mandatory step to build a prognosis method but is, in itself, not sufficient. The most common methodology is to perform a multivariate Cox regression on a selected few miRNAs and use the Cox coefficients to build a risk score [11, 17–19]. Additionally, we assessed the accuracy of a prognosis method through the Concordance index (c-index), a standard approach that computes the rank concordance between a risk value and the survival time and avoids thus the arbitrary stratification of patients into high- and low-survival groups [20–22]. Building on that, we identified 5 miRNAs, representative of the previously identified 5 miRNA clusters, that are best able to predict overall survival in the training cohort. That is miR-21, miR-10b, miR-143, miR-183, and miR-192, which will be further referred to as the “top miRs” signature. All of them have been reported to play a role in cell proliferation or metastasis but miR-192 and miR-183 have never been included in a miRNA signature for ccRCC. In that respect, miR-192 is of primary interest as it has been well studied but never reported as a potential biomarker for ccRCC. A correlation analysis on gene expression confirmed the regulation of miR-192 by *HNF1A* (Pearson's rho of 0.59) as reported by Khella et al. [23] however proposed targets, such as *FZD6*, show a weak correlation in the TCGA data (Pearson's rho of −0.31). Among the top 5 negative correlation in the training cohort, only *TPM4* (tropomyosin 4, Pearson's rho of −0.43) and *DBN1* (drebrin 1, Pearson's rho of −0.42) had a potential 3′-UTR binding reported by miRWalk2.0 [24]. Both proteins have relations with the actin cytoskeleton [25, 26], which strengthens the hypothesis that miR-192 is involved in the epithelial-mesenchymal transition and metastases [23, 27].

**Figure 2:**
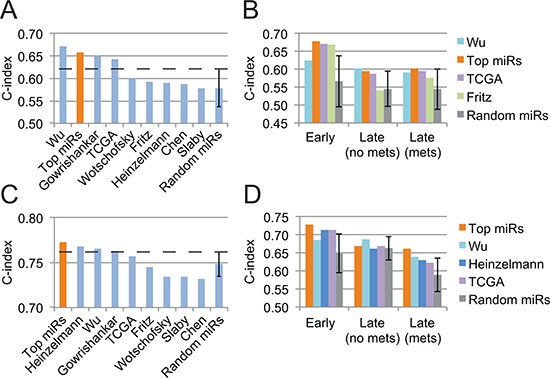
Performance assessment of several prognosis signatures on the validation cohort **(A)** Overall survival prediction from pure miRNA signatures. **(B)** Detailed analysis, stratified by tumor stage, of miRNA signatures that outperform a random 5-miRNA signature by more than a standard deviation. **(C)** Prognosis performance of miRNA signatures when combined with the patient's age and TNM tumor stage. **(D)** Same as B but for miRNA signature combined with the patient's age and tumor stage. Error bars represent the standard deviation.

When compared to other published miRNA signatures on the validation cohort, our “top miRs” signature outperforms all other, to the exception of Wu *et al*.'s, in terms of overall survival prediction (Fig. 2A). Of note, it surpassed all other signatures in the training cohort (Supplementary Fig. S1A). However, with respect to tumor stage and metastasis, one observes that our “top miRs” signature is superior to all other signature for early-stage patients and for late-stage patients with metastases, and only slightly inferior to Wu *et al*.'s for late-stage patients without metastases (Fig. 2B). Interestingly, only four signatures, including ours, lie further away than one standard deviation from a random 5-miRNA signature.

We then combined clinical features with our miRNA signature to increase its prognosis power. Through a model selection procedure, we identified the TNM stage and the patient's age as the most informative features to be included. The new composite signature allowed an increase of 0.11 in the c-index (from 0.66 to 0.77), which places it as the best signature for OS prognostication on the validation cohort (Fig. 2C) and second best on the training cohort (Supplementary Fig. S1B). Wu *et al*.'s signature, which ranks first when using miRNAs only, did not benefit as much from the addition of clinical variables. Finally, our composite signature is the only one to be largely superior to a random miRNA signature complemented with age and stage for early-stage patients and late-stage patients with metastases (Fig. 2D).

### miRisk5 describes a clinically-applicable risk

**Figure 3:**
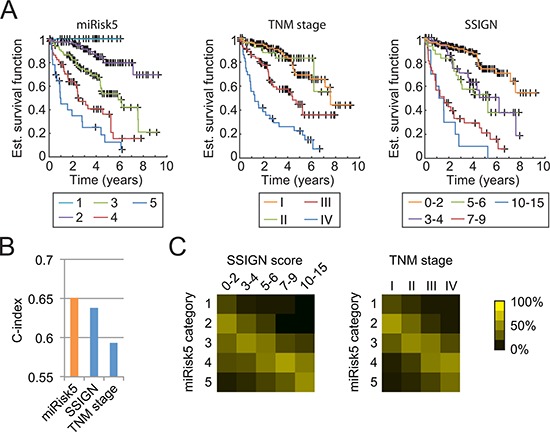
Comparison of miRisk5 with the TNM staging system and the Mayo Clinic score (SSIGN) on the validation cohort **(A)** Kaplan-Meier curves for each risk category. **(B)** Prognosis performance of the three methods. **(C)** Graphical visualization of confusion matrices for risk categories between miRisk5 and the two other clinical scores. Values are normalized column-wise by the respective total number of patients.

A continuous risk, as resulting from a Cox regression analysis, has a high discriminative power but is unpractical for clinical usage. Consequently, we summarized our composite risk into 5 categories to provide a usable tool, referred to as miRisk5 (see Supplementary Methods). Kaplan-Meier survival curves, as displayed in Fig. 3A, show that our summarized risk is able to span a wider range of patient outcomes than the TNM staging or the Mayo Clinic score (SSIGN), which is another well-established scoring system for RCC patients [28]. Both the TNM staging and the SSIGN score identify three different groups while miRisk5 clearly separates 5 different groups. Additionally, miRisk5 provides a substantial amelioration with respect to TNM staging (c-index of 0.65 vs. 0.59 for the TNM staging; Fig. 3B). Although it does not significantly outperform the SSIGN score in terms of prognosis, it does assign patients into different risk groups than the SSIGN or the TNM score (Fig. 3C).

### The 5-miRNA prognosis signature is specific to ccRCC

**Figure 4:**
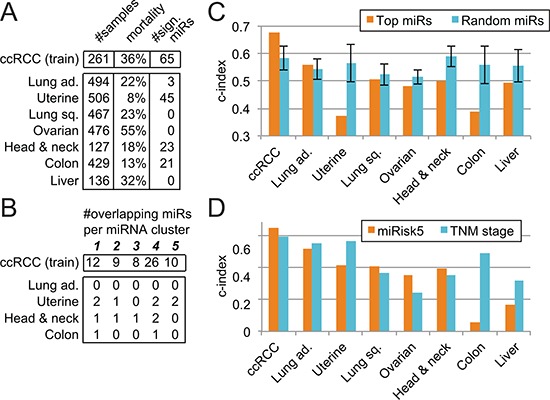
Evaluation of the 5-miRNA signature across 7 other cancer types **(A)** Identification of miRNAs associated to overall survival. **(B)** Overlap between the results in other cancer types and the 65 miRNAs identified in ccRCC (training cohort) and their respective clusters. **(C)** Prognosis comparison of our pure 5-miRNA signature with respect to a random signature in several cancers. Error bars represent the standard deviation. **(D)** Performance evaluation of the miRisk5 signature vs. the TNM staging system alone in diverse cancer types.

We extended this type of analysis to other cancer types represented in the TCGA database and we selected 7 cancer types where genomic information on a considerable number of tumors was available. We applied the same approach as outlined before for ccRCC to identify miRNAs in each cancer type that correlate with overall survival (Supplementary Table S2). This analysis revealed to our surprise that some cancers, including ovarian, liver, and lung cancer, display zero or only a few miRNAs associated to overall survival while others, such as ccRCC and uterine cancer, demonstrate a high number of miRNAs correlating with overall survival (Fig. 4A). A re-analysis of the ovarian cancer data using the same methodology as the TCGA Research Network, that is a univariate Cox regression analysis, led to the same conclusion.

Next we used these results in a comparative analysis to assess whether any of these miRNAs overlap with the ones identified in ccRCC and their associated clusters. No other cancer type displayed a similar pattern of OS-associated miRNAs (Fig. 4B). Uterine carcinoma and head and neck carcinoma have overlapping miRNAs in four out of five clusters but only 3 of them are cluster representatives: miR-10b for head and neck and miR-146b-3p and miR-221 for uterine.

Prognostication of overall survival based on our 5-miRNA signature in other cancer types unveiled the specificity of our signature to ccRCC (Fig. 4C). In none of the other cancer types is our 5-miRNA signature able to surpass a random 5-miRNA signature. The use of our composite signature, miRisk5, yields similar results for lung adenocarcinoma, uterine, colon, and liver cancers but an improvement for lung squamous, ovarian, and head and neck cancers (Fig. 4D). From Fig. 4C, we know that the 5-miRNA signature is uninformative, hence the observed improvements are likely due to the combination of the patient's age with the pathological stage.

### Subgroups of patients characterized by the 5-miRNA signature demonstrate different outcome, pathological stages and ccRCC cancer gene alterations

**Figure 5:**
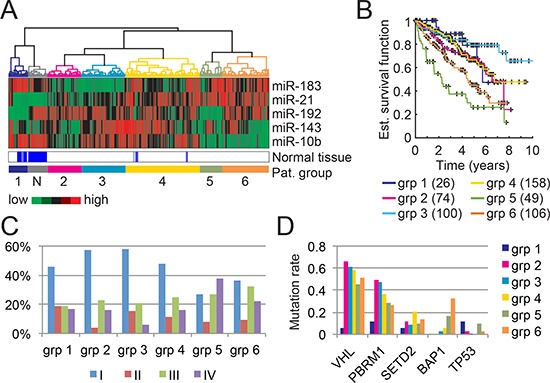
Patient clusters and associated characteristics based on our 5-miRNA signature **(A)** Clustering of 513 ccRCC tumor samples and 71 normal kidney samples into 7 clusters based on our 5-miRNA signature. **(B)** Kaplan-Meier curves of each patient clusters (tumor samples only). The number of patients in each cluster is indicated in parentheses. **(C)** Tumor stage distribution with respect to the patient clusters. **(D)** Mutation rates of well-known tumor suppressor genes in ccRCC with respect to each patient cluster.

To investigate patient characteristics inherent to the expression levels of our 5-miRNA signature, we performed a clustering analysis on the complete ccRCC cohort (513 primary tumor samples and 71 normal tissue samples) and identified 7 major patient clusters (Fig. 5A). As expected, all normal samples were clustered together. Of note, there seems to be two groups of normal samples (group “N” and part of group 1) that differ in in their miR-192 and miR-183 expression levels. Patients with normal samples in group “N” are mostly in early stage (51%) while patients with normal samples in group 1 are principally in late stage (70%). However, there was no correlation in miR-192 levels between normal and tumor tissue in matched samples.

Kaplan-Meier curves, as displayed in Fig. 5B, reveal that patients with high miR-21 and low miR-10b levels have a worst survival (i.e. patient groups 5 and 6), an observation that is consistent with the findings of Fritz *et al*. [12]. As expected, these two groups are also enriched in late-stage tumors (Fig. 5C). Despite their similarities, group 5 differs from group 6 by a higher number of stage IV patients (38% vs 22%), a worse survival, and low miR-192 expression levels. Interestingly, miR-192 expression does not significantly correlate with tumor stage (Wilcoxon test between early and late stage patients) and hence might be a marker for two different ccRCC subtypes. A recent study revealed the existence of two ccRCC subtypes with different outcomes and a panel of 34 genes to differentiate them [29]. miR-192 expression levels highly correlate with their subtype classification (Wilcoxon test *p*-value: 1.95×10-11) and could also act as a subtype predictor (area under ROC curve of 0.7).

We then assessed whether the identified patient clusters feature distinct genetic alterations in known ccRCC cancer genes. Recent comprehensive genomic analyses of ccRCC revealed in addition to *VHL* mutations, recurrent mutations in several other ccRCC tumor suppressor genes including *PBRM1*, *SETD2*, and *BAP1* [16, 30]. The products of these genes have been implicated in chromatin regulation but their precise functions in ccRCC development remain to be elucidated. Our analysis of these four most frequently mutated genes in ccRCCs revealed enrichment in *BAP1* mutations for patient groups 5 and 6, but no distinct patterns for *SETD2* (Fig. 5D). *BAP1* mutations are known to correlate with poor survival [31] and hence could explain the low survival of patients from group 5. Nonetheless, patients from group 6, despite having the highest *BAP1* mutation rate, display a better survival than patients from group 5 (median survival of 52.1 months vs. 25.6 months for group 5). One can thus hypothesize that *BAP1* mutations are associated to late-stage tumors but not to overall survival. Also, a recent study showed that *BAP1* and *SETD2* mutations tend to appear late in tumor development [3], a finding that supports our observation.

The miR-192/194 cluster has been reported to depend on the p53 mutation status in multiple myeloma, hepatocellular, and renal diseases but this association has not been studied in RCC [32, 33]. Here, we observe that the two patient groups characterized by low miR-192 expression levels (group 1 and group 5) also display a higher p53 mutation rate than the other groups (Fisher exact test *p*-value: 0.0022) hence linking the regulation of miR-192 by p53 to ccRCC.

### BAP1 mutations are associated to tumor progression but not with overall survival

**Figure 6:**
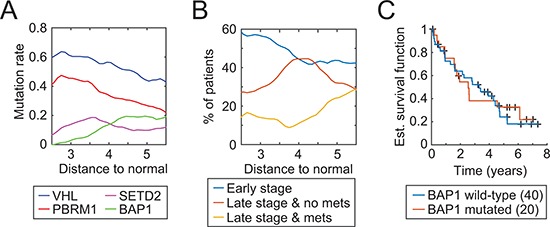
Tumor progression as measured by the 5-miRNA signature and associated characteristics **(A)** Evolution of mutation rates of four well-known tumor suppressor genes with respect to miRNA levels of normal kidney samples. **(B)** Distribution of tumor stage and metastasis as a function of our 5-miRNA distance **(C)** Kaplan-Meier curves with respect to the BAP1 mutation status of late-stage patients with a distance greater than 4.5.

To further investigate a potantial association of *BAP1* mutation and tumor stage, we measured tumor progression as the distance to the average miRNA values of normal patients (group “N”) and analyzed the mutation rates of the four most mutated genes in ccRCC with respect to the aforementioned distance. We observed that the *BAP1* mutation rate increases with the distance, as one would expect, although not in a linear fashion (Fig. 6A). Indeed, the gain in *BAP1* mutations seems to be restricted to patients within a distance of 3.5 to 4.5 to the normal group. *VHL* and *PBRM1* mutations decrease linearly as the distance augments and *SETD2* mutations peak at a distance of 3.5. *VHL* and *PBRM1* have been reported as founding mutations [3] and high mutation rates at short distances concur with this hypothesis. As the tumor progresses, the environmental pressure changes and core mutations may not be required anymore, which in turn would translate into a decrease in mutation rates.

A comparison of pathological stage with our progression distance reveals a similar picture (Fig. 6B). Short distances correlate with early stage while metastases occur only at a distance of 4 or greater. Late-stage tumors without metastases peak around 4 then diminish, in agreement with the hypothesis that a distance based on our 5-miRNA signature is reflective of the tumor evolution. One can thus deduce a sequential order of core mutations in ccRCC: *VHL* and *PBRM1* (founding mutations), *SETD2* (transitional mutation), and finally *BAP1* (metastasis).

*BAP1* mutations are related to tumor progression but do not define a category of patient with a worse outcome. As seen on Fig. 6C, a comparison of *BAP1* wild-type vs. mutated late-stage patients with a distance greater than 4.5 (the point where the BAP1 mutation rate stops to increase) shows no significant difference in overall survival. A prognosis based on our 5-miRNA signature can consequently better capture tumor progression and a patient outcome in ccRCC than a mutation-based prognosis.

## DISCUSSION

The publication of large-scale datasets on human ccRCC has dramatically changed our perspective of the genomic landscape of this cancer type and offered new opportunities for better prognostication of ccRCC, prediction of response to molecular-targeted therapy, and development of novel, more effective therapies. In this report we performed an integrated analysis of multi-dimensional data from the TCGA archive (584 ccRCC cases split into similarly-sized training and validation sets) and identified a novel 5-miRNA signature able to classify ccRCC in distinct subgroups and to function as molecular descriptors for ccRCC prognostication [34]. Interestingly, our unique miRNA signature allowed also the identification of six distinct patient clusters that span different clinical outcomes, pathological features, and somatic mutation profiles. An analysis of the distinct characteristics of each cluster led to new insights regarding the role of *BAP1* mutations in ccRCCs. That is, enrichment in *BAP1* mutations is associated with late tumor stages but does not define *per se* a patient's outcome.

Transcriptome signatures represent an important basis for the development of biomarkers and their application in clinical settings and must thus display high reliability and robustness. The TCGA Research Network, who disclosed the ccRCC cohort, performed a global analysis with a focus on genetic lesions and changes in miRNA and gene expression levels. The difference in methodology and thresholds between our approach and the one used by the TCGA Research Network—log-rank test vs. univariate Cox regression—revealed substantial differences in terms of deduced signatures. As miRNAs are known to affect gene regulation in a non-linear fashion [35], a log-rank test approach, as used here, appears to be more appropriate.

Insights into the biology of ccRCC have provided rationales for treating the disease. Although recent advances have improved patient outcomes, targeted agents still display low response rates and are not without toxic side effects. Accurate ccRCC subtyping is therefore imperative and the molecular signature described here represent a reliable foundation for the development of molecular biomarkers of ccRCC facilitating stratification of patients and prognosticating disease outcome.

## METHODS

### Data

584 clear-cell renal cell carcinoma samples from distinct patients were collected from the TCGA online database (http://tcga-data.nci.nih.gov, accessed on October 28, 2014). Samples comprise miRNA- and RNA-sequencing data, clinical information, and mutation status of several genes. For more details regarding the TCGA cohort, please refer to the original publication [16].

Clinical data and miRNA expression levels were also analyzed for uterine corpus endometrial carcinoma (540 samples), ovarian serous cystadenocarcinoma (484 samples), lung adenocarcinoma (542 samples), lung squamous cell carcinoma (512 samples), colon adenocarcinoma (438 samples), head and neck squamous cell carcinoma (463 samples), and hepatocellular carcinoma (186 samples). Only primary tumor samples were considered for the analysis. Other cancer types available in the TCGA had either too few primary tumor samples with miRNA-sequencing data or a too low mortality rate within the cohort.

Training and validation sets were created from the ccRCC cohort based on the miRNA-sequencing platform. This resulted in a 323-sample training cohort (252 primary tumor samples and 71 normal tissue samples, analyzed by Illumina HiSeq) and a 261-sample validation cohort (all primary tumors, analyzed by Illumina GenomeAnalyzer). No bias in tumor stage, age, overall survival, or gender distribution was observed between the training and validation cohorts. Both training and validation cohorts contain samples from several institutions (mainly from Memorial Sloane Kettering Cancer Center, University of Pittsburgh, MD Anderson Cancer Center, Harvard Cancer Center, and International Genomics Consortium).

### Statistical and computational analyses

#### miRNA and mRNA expression values

TCGA miRNA isoform expression values (level 3) were summed to obtain strand-specific expression values (miRBase v16). Gene expression levels were obtained from RNA-sequencing data (level 3). All samples were normalized by the Upper Quartile method [36].

#### miRNA signature identification

For each miRNA in the training cohort, two groups of patients were constructed based on expression levels of the miRNA: Lower than the 25% quartile and higher than the 75% quartile. A log-rank test was then applied to determine if the difference in terms of overall survival between the two groups was significant. All miRNAs with a false discovery rate below 0.1 and a third quartile value above 10 RPKM were defined as significantly associated to overall survival (OS).

Clustering of significant OS-associated miRNAs was performed through a non-negative matrix factorization (NMF) with ranks tested from 2 to 15 [37]. Representative miRNAs for each cluster were selected based on their basis coefficient (> 0.75). All possible combinations of representative miRNAs (such that to have only one representative per cluster) were tested to obtain the “top 5-miRNA” signature. A multivariate Cox regression analysis on miRNA expression values was used to compute a risk for each combination. The addition of clinical variables was assessed through a forward model selection procedure (Aikake information criterion) combined with a Cox regression. Only clinical variables where at least three quarters of the patients in the training cohort had an assigned value were considered: gender, age, pathological stage, presence of metastasis, Fuhrman grade, TNM stage, hemoglobin level, platelet level, and white cell count. A bootstrapping procedure (resampling with replacement) was performed to test the robustness of the results (100 iterations, bootstrap value > 0.8).

For each signature, coefficients from a multivariate Cox regression analysis on the training cohort were used to compute a risk on the validation cohort. Performance was assessed through the concordance index (c-index). To test the significance of the results, a random miRNA signature was designed by selecting 5 random miRNAs from the TCGA dataset. Sampling was performed over 1000 iterations to obtain an average C-index and its standard deviation. In the pan-cancer analysis, Cox coefficients for the random miRNA signature were inferred within a 10-fold cross-validation framework. Cox coefficients obtained from the training cohort in ccRCC were used to compute the miRisk5 value in other cancer types.

#### miRisk5

The discrete risk score, miRisk5, is computed as follow.

$$miRisk5=\left\{ \begin{array}{l} 1\;if\;r\leq 1\\ 2\;if\;r>1\;and\;r\leq 2\\ 3\;if\;r>2\;and\;r\leq 3\\ 4\;if\;r>3\;and\;r\leq 4\\ 5\;if\;r>4\; \end{array}\right.$$

where $r=0.22\times v_{\text{miR-}21}-0.38\times v_{\text{miR-}143}-0.072\times v_{\text{miR-}10\text{b}}-4.26\times v_{\text{miR-}192}-3.57\times v_{\text{miR-}183}\;+27\times \text{age}+637\times \text{stage}$ and *v_m_* is the normalized read count for miRNA *m* (upper quartile method).

#### Patient clustering

Hierarchical clustering with Ward linkage was performed on log2 miRNA expression values of the complete ccRCC cohort (training plus validation). Patient clusters were defined through a cutoff value of 15. Distance to the normal patient group was computed as an Euclidean distance on log2 miRNA expression values, weighted by the respective standard deviations. Characteristics curves (mutation and stage) were computed through a sliding window of 50 patients with respect to the distance. Displayed curves were smoothened by a moving average of ± 0.5.

## SUPPLEMENTARY FIGURE AND TABLES



## References

[1] American Cancer Society (2012). Cancer Facts & Figures 2012. Atlanta Am Cancer Soc.

[2] Zhang L, Xu B, Chen S, Lu K, Liu C, Wang Y, Zhao Y, Zhang X, Liu D, Chen M (2013). The complex roles of microRNAs in the metastasis of renal cell carcinoma. J. Nanosci. Nanotechnol.

[3] Gerlinger M, Horswell S, Larkin J, Rowan AJ, Salm MP, Varela I, Fisher R, McGranahan N, Matthews N, Santos CR, Martinez P, Phillimore B, Begum S, Rabinowitz A, Spencer-Dene B, Gulati S, Bates PA, Stamp G, Pickering L, Gore M, Nicol DL, Hazell S, Futreal PA, Stewart A, Swanton C (2014). Genomic architecture and evolution of clear cell renal cell carcinomas defined by multiregion sequencing. Nat. Genet.

[4] Linehan WM, Rathmell WK (2012). Kidney cancer. Urol. Oncol. Elsevier Inc.

[5] Edge SB, Compton CC (2010). The American Joint Committee on Cancer: the 7th edition of the AJCC cancer staging manual and the future of TNM. Ann. Surg. Oncol.

[6] Schoof C (2012). MicroRNAs in cancer treatment and prognosis. Am J Cancer Res.

[7] Chen J, Zhang D, Zhang W (2013). Clear cell renal cell carcinoma associated microRNA expression signatures identified by an integrated bioinformatics analysis. J. Transl. Med. Journal of Translational Medicine.

[8] Heinzelmann J, Henning B (2011). Specific miRNA signatures are associated with metastasis and poor prognosis in clear cell renal cell carcinoma. World J. Urol.

[9] Slaby O, Redova M (2012). Identification of microRNAs associated with early relapse after nephrectomy in renal cell carcinoma patients. Genes. Chromosomes Cancer.

[10] Wotschofsky Z, Busch J, Jung M (2012). Diagnostic and prognostic potential of differentially expressed miRNAs between metastatic and non-metastatic renal cell carcinoma at the time of nephrectomy. Clin. Chim. Acta.

[11] Wu X, Weng L, Li X, Guo C, Pal S, Jin J (2012). Identification of a 4-microRNA signature for clear cell renal cell carcinoma metastasis and prognosis. PLoS One.

[12] Fritz HKM, Lindgren D, Ljungberg B, Axelson H, Dahlbäck B (2014). The miR(21/10b) ratio as a prognostic marker in clear cell renal cell carcinoma. Eur J Cancer.

[13] Gowrishankar B, Ibragimova I, Zhou Y, Slifker MJ, Devarajan K, Al-Saleem T, Uzzo RG, Cairns P (2014). MicroRNA expression signatures of stage, grade, and progression in clear cell RCC. Cancer Biol. Ther.

[14] Petillo D, Kort EJ, Anema J, Furge KA, Yang XJ (2009). MicroRNA profiling of human kidney cancer subtypes.

[15] Binder H, Porzelius C, Schumacher M (2011). An overview of techniques for linking high-dimensional molecular data to time-to-event endpoints by risk prediction models. Biometrical J.

[16] The Cancer Genome Atlas Research Network (2013). Comprehensive molecular characterization of clear cell renal cell carcinoma. Nature.

[17] Zhang W, Zhang J, Yan W, You G, Bao Z, Li S, Kang C, Jiang C, You Y, Zhang Y, Chen CC, Song SW, Jiang T (2013). Whole-genome microRNA expression profiling identifies a 5-microRNA signature as a prognostic biomarker in chinese patients with primary glioblastoma multiforme. Cancer.

[18] Han Z, Zhong L, Teng M, Fan J, Tang H (2012). Identification of recurrence-related microRNAs in hepatocellular carcinoma following liver transplantation. Mol. Oncol. Elsevier B.V.

[19] Shih KK, Qin L-X, Tanner EJ, Zhou Q, Bisogna M, Dao F, Olvera N, Viale A, Barakat RR, Levine D a (2011). A microRNA survival signature (MiSS) for advanced ovarian cancer. Gynecol. Oncol. Elsevier Inc.

[20] Harrell F, Lee K, Mark D (1996). Multivariable prognostic models: issues in developing models, evaluating assumptions and adequacy, and measuring and reducing errors. Stat Med.

[21] Kattan MW (2006). Validating a prognostic model. Cancer.

[22] Margolin Aa, Bilal E, Huang E, Norman TC, Ottestad L, Mecham BH, Sauerwine B, Kellen MR, Mangravite LM, Furia MD, Vollan HKM, Rueda OM, Guinney J, Deflaux Na, Hoff B, Schildwachter X, Russnes HG, Park D, Vang VO, Pirtle T, Youseff L, Citro C, Curtis C, Kristensen VN, Hellerstein J, Friend SH, Stolovitzky G, Aparicio S, Caldas C, Børresen-Dale A-L (2013). Systematic analysis of challenge-driven improvements in molecular prognostic models for breast cancer. Sci. Transl. Med.

[23] Khella HWZ, Bakhet M, Allo G, Jewett M a S, Girgis a H, Latif a, Girgis H, Von Both I, Bjarnason G a, Yousef GM (2013). miR-192, miR-194 and miR-215: a convergent microRNA network suppressing tumor progression in renal cell carcinoma. Carcinogenesis.

[24] Dweep H, Sticht C, Pandey P, Gretz N (2011). miRWalk— database: prediction of possible miRNA binding sites by “walking” the genes of three genomes. J Biomed. Inform. Elsevier Inc.

[25] Peitsch WK, Grund C, Kuhn C, Schnölzer M, Spring H, Schmelz M, Franke WW (1999). Drebrin is a widespread actin-associating protein enriched at junctional plaques, defining a specific microfilament anchorage system in polar epithelial cells. Eur J Cell Biol.

[26] Häbig K, Gellhaar S, Heim B, Djuric V, Giesert F, Wurst W, Walter C, Hentrich T, Riess O, Bonin M (2013). LRRK2 guides the actin cytoskeleton at growth cones together with ARHGEF7 and Tropomyosin 4. Biochim. Biophys. Acta Elsevier B.V.

[27] Hong J-P, Li X-M, Li M-X, Zheng F-L (2013). VEGF suppresses epithelial-mesenchymal transition by inhibiting the expression of Smad3 and miR-192, a Smad3-dependent microRNA. Int J. Mol. Med.

[28] Zigeuner R, Hutterer G, Chromecki T, Imamovic A, Kampel-Kettner K, Rehak P, Langner C, Pummer K (2010). External validation of the Mayo Clinic stage, size, grade, and necrosis (SSIGN) score for clear-cell renal cell carcinoma in a single European centre applying routine pathology. Eur. Urol.

[29] Brooks SA, Brannon AR, Parker JS, Fisher JC, Sen O, Kattan MW, Hakimi AA, Hsieh JJ, Choueiri TK, Tamboli P, Maranchie JK, Hinds P, Miller CR, Nielsen ME, Rathmell WK (2014). ClearCode34: a prognostic risk predictor for localized clear cell renal cell carcinoma. Eur. Urol. European Association of Urology.

[30] Sato Y, Yoshizato T, Shiraishi Y, Maekawa S, Okuno Y, Kamura T, Shimamura T, Sato-Otsubo A, Nagae G, Suzuki H, Nagata Y, Yoshida K, Kon A, Suzuki Y, Chiba K, Tanaka H, Niida A, Fujimoto A, Tsunoda T, Morikawa T, Maeda D, Kume H, Sugano S, Fukayama M, Aburatani H, Sanada M, Miyano S, Homma Y, Ogawa S (2013). Integrated molecular analysis of clear-cell renal cell carcinoma. Nat. Genet.

[31] Brugarolas J (2013). PBRM1 and BAP1 as novel targets for renal cell carcinoma. Cancer J.

[32] Kruetzfeldt J, Roesch N, Hausser J, Manoharan M, Zavolan M, Stoffel M (2012). MicroRNA-194 Is a Target of Transcription Factor 1 (Tcf1, HNF1 a) in Adult Liver and Controls Expression of Frizzled-6. Hepatology.

[33] Deshpande SD, Putta S, Wang M, Lai JY, Bitzer M, Nelson RG, Lanting LL, Kato M, Natarajan R (2013). Transforming growth factor-β-induced cross talk between p53 and a microRNA in the pathogenesis of diabetic nephropathy. Diabetes.

[34] McShane LM, Altman DG, Sauerbrei W, Taube SE, Gion M, Clark GM (2005). Reporting recommendations for tumor marker prognostic studies (REMARK). J Natl. Cancer Inst.

[35] Nissan T, Parker R (2008). Computational analysis of miRNA-mediated repression of translation: implications for models of translation initiation inhibition. RNA.

[36] Dillies M-A, Rau A, Aubert J, Hennequet-Antier C, Jeanmougin M, Servant N, Keime C, Marot G, Castel D, Estelle J, Guernec G, Jagla B, Jouneau L, Laloë D, Le Gall C, Schaëffer B, Le Crom S, Guedj M, Jaffrézic F (2012). A comprehensive evaluation of normalization methods for Illumina high-throughput RNA sequencing data analysis. Brief. Bioinform.

[37] Gaujoux R, Seoighe C (2010). A flexible R package for nonnegative matrix factorization. BMC Bioinformatics.

