# Supplementary material for: Integrated genomic analysis identifies subclasses and prognosis signatures of kidney cancer
Source: Oncotarget. 2015 Mar 24;6(12):10521–31. doi: 10.18632/oncotarget.3294 (PMC4496372; doi:10.18632/oncotarget.3294)
Supplement: Supplementary file 1 [file oncotarget-06-10521-s001.pdf]

SUPPLEMENTARY FIGURE AND TABLES

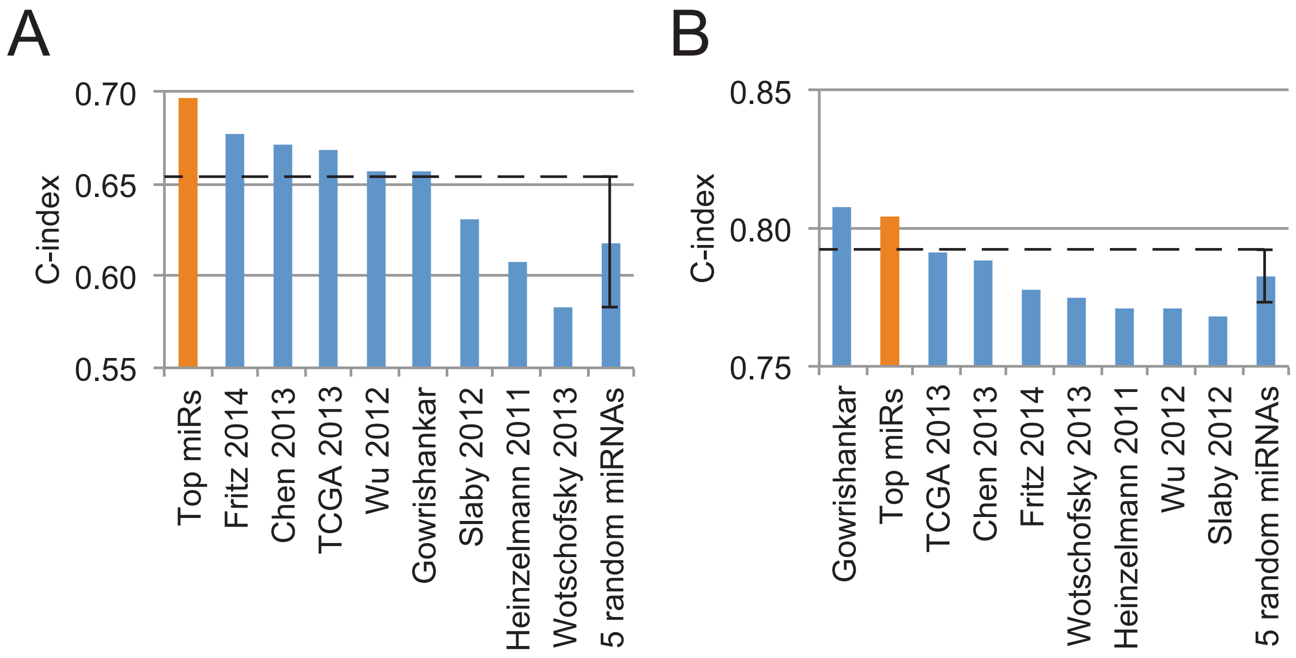

Supplementary Figure S1: Performance assessment of several prognosis signatures on the training cohort. A. Overall survival prediction from pure miRNA signatures. **B.** Prognosis performance of miRNA diverse signatures when combined with the patient's age and tumor TNM stage.

Supplementary Table S1. List of miRNAs significantly associated to overall survival in ccRCC. miRNAs representative of a NMF cluster are highlighted in bold.

	training cohort				validation cohort
**miRNA**	**NMF clusters**	**avg RPKM**	**log-rank test *p*-value**	**pFDR**	**log-rank test *p*-value**
miR-92b	1	53.2	0.0000	0.0019	0.0194
**miR-146b-5p**	1	638.3	0.0001	0.0023	0.0131
miR-365	1	692.4	0.0005	0.0078	0.0225
b	1	158305.5	0.0004	0.0080	0.0425
miR-425	1	92.3	0.0020	0.0164	0.0410
miR-21*	1	1235.7	0.0025	0.0169	0.0388
miR-193a-3p	1	9.0	0.0042	0.0227	0.6545
**miR-146b-3p**	1	50.3	0.0047	0.0236	0.1078
miR-223	1	45.2	0.0054	0.0254	0.0001
**miR-155**	1	528.5	0.0278	0.0745	0.0530
miR-142-5p	1	2060.7	0.0308	0.0756	0.1086
miR-224	1	28.7	0.0329	0.0794	0.9109
miR-139-3p	2	20.4	0.0074	0.0324	0.0007
miR-27a*	2	8.1	0.0116	0.0443	0.0593
miR-195	2	52.4	0.0137	0.0484	0.2113
**miR-1**	2	23.8	0.0134	0.0486	0.6372
miR-145*	2	42.3	0.0164	0.0552	0.2761
let-7c	2	52.8	0.0219	0.0660	0.6019
miR-584	2	42.7	0.0305	0.0761	0.0047
miR-181c	2	26.1	0.0404	0.0927	0.4785
**miR-143**	2	78398.9	0.0417	0.0942	0.1798
miR-27b*	3	16.6	0.0006	0.0081	0.0000
miR-27b	3	18.6	0.0014	0.0133	0.0004
miR-139-5p	3	134.3	0.0016	0.0135	0.0035
miR-10b*	3	43.0	0.0034	0.0215	0.0000
miR-24-1*	3	17.4	0.0041	0.0227	0.0029
**miR-10b**	3	203997.4	0.0044	0.0228	0.0005
miR-106a	3	10094.6	0.0079	0.0337	0.9325
miR-125a-5p	3	1065.2	0.0229	0.0663	0.0416
miR-204	4	520.0	0.0000	0.0015	0.0316
miR-101	4	11360.8	0.0000	0.0017	0.0001
let-7b	4	11862.2	0.0000	0.0023	0.0480

(*Continued*)

	training cohort				validation cohort
**miRNA**	**NMF clusters**	**avg RPKM**	**log-rank test *p*-value**	**pFDR**	**log-rank test *p*-value**
miR-301a	4	7.7	0.0003	0.0056	0.0514
miR-1307	4	50.2	0.0006	0.0083	0.5439
**miR-194***	4	98.8	0.0008	0.0091	0.0004
let-7b*	4	25.2	0.0021	0.0151	0.5652
miR-2355-5p	4	56.7	0.0032	0.0212	0.0050
miR-1468	4	12.2	0.0037	0.0221	0.0095
miR-190	4	12.3	0.0039	0.0225	0.0000
**miR-192***	4	24.6	0.0056	0.0255	0.0324
miR-23b	4	1254.8	0.0086	0.0354	0.0001
miR-99b	4	39179.7	0.0120	0.0446	0.0005
miR-455-5p	4	89.2	0.0138	0.0476	0.0206
miR-181a	4	1461.9	0.0175	0.0561	0.0218
**miR-192**	4	10146.0	0.0191	0.0599	0.0061
miR-590-5p	4	11.8	0.0229	0.0675	0.8702
miR-15b	4	128.6	0.0245	0.0683	0.4587
miR-151-5p	4	86.5	0.0244	0.0691	0.9245
miR-576-5p	4	11.6	0.0270	0.0738	0.9911
miR-126	4	15.4	0.0281	0.0739	0.0485
miR-653	4	110.1	0.0300	0.0760	0.1164
miR-1269	4	257.1	0.0298	0.0771	0.0053
miR-99a	4	683.5	0.0336	0.0784	0.3016
miR-542-3p	4	400.5	0.0333	0.0791	0.8851
miR-423-3p	4	99.5	0.0440	0.0980	0.1079
**miR-221**	5	1071.4	0.0002	0.0049	0.1200
miR-22*	5	19.4	0.0008	0.0086	0.1403
**miR-183**	5	1212.7	0.0014	0.0129	0.1588
**miR-182**	5	4261.6	0.0013	0.0135	0.2661
miR-29b	5	578.5	0.0021	0.0158	0.0188
miR-222	5	115.1	0.0047	0.0228	0.0834
miR-335*	5	199.2	0.0098	0.0395	0.0268
miR-200a*	5	17.2	0.0113	0.0440	0.1788
miR-148a*	5	11.1	0.0172	0.0567	0.9594
miR-193b	5	42.0	0.0210	0.0647	0.0191

Supplementary Table S2. Pan-cancer analysis of overall survival associated miRNAs. No miRNAs significantly associated to overall survival were identified for lung squamous carcinoma, ovarian serous carcinoma, and hepatocellular carcinoma.

COAD		HNSC		LUAD		UCEC	
**miRNA**	**pFDR**	**miRNA**	**pFDR**	**miRNA**	**pFDR**	**miRNA**	**pFDR**
let-7g*	0.0001	miR-337-3p	0.0090	miR-212	0.0362	miR-24	0.0083
miR-16-2*	0.0035	miR-127-3p	0.0132	miR-582-3p	0.0390	miR-146b-3p	0.0107
miR-3647-3p	0.0038	miR-654-3p	0.0155	miR-582-5p	0.0405	miR-106b*	0.0114
miR-16	0.0043	miR-148b	0.0306			miR-100	0.0149
miR-126*	0.0120	miR-493*	0.0316			miR-324-3p	0.0163
let-7d	0.0130	miR-409-3p	0.0316			miR-1269	0.0167
miR-3607-3p	0.0131	miR-125b-2*	0.0357			miR-628-5p	0.0175
miR-409-5p	0.0315	miR-379	0.0363			miR-107	0.0179
miR-425*	0.0319	miR-134	0.0368			miR-31	0.0225
T0006766	0.0372	miR-382	0.0430			miR-25	0.0231
miR-148a	0.0416	miR-136*	0.0447			miR-335*	0.0236
miR-33a	0.0562	miR-758	0.0467			miR-181b	0.0241
miR-200c*	0.0659	miR-181c	0.0529			miR-92a	0.0248
miR-29c*	0.0701	miR-16-2*	0.0645			miR-17*	0.0252
miR-3130-5p	0.0751	miR-10b	0.0688			miR-17	0.0266
T0005910	0.0771	miR-23b	0.0725			miR-18a	0.0267
miR-148b	0.0799	miR-142-5p	0.0729			miR-425*	0.0284
miR-193a-3p	0.0805	miR-26a	0.0759			miR-330-5p	0.0293
miR-455-3p	0.0922	miR-146a	0.0817			miR-652	0.0400
miR-126	0.0939	miR-127-5p	0.0817			let-7g	0.0431
miR-3653	0.0988	miR-142-3p	0.0821			miR-361-3p	0.0469
		miR-99a	0.0842			miR-99a	0.0471
		miR-148a	0.0846			miR-320b	0.0477
						miR-125b-2*	0.0539
						miR-126*	0.0574
						miR-379	0.0600
						miR-29a	0.0645
						miR-148b	0.0653
						miR-449a	0.0658
						let-7c	0.0666
						miR-128	0.0671

(*Continued*)

COAD		HNSC		LUAD		UCEC	
miRNA	pFDR	miRNA	pFDR	miRNA	pFDR	miRNA	pFDR
						miR-361-5p	0.0675
						miR-31*	0.0681
						miR-103	0.0683
						miR-20a	0.0731
						miR-106b	0.0828
						miR-21*	0.0838
						miR-149	0.0842
						miR-193a-5p	0.0868
						miR-125b	0.0877
						let-7i*	0.0880
						miR-3200-3p	0.0900
						miR-324-5p	0.0902
						miR-1180	0.0917
						miR-221	0.0978
